# How to address the inverse care law and increase GP recruitment in areas of socioeconomic deprivation: a qualitative study of GP trainees’ views and experiences in the UK

**DOI:** 10.3399/BJGPO.2023.0201

**Published:** 2024-05-01

**Authors:** Matthew J Armstrong, Josephine M Wildman, Sarah Sowden

**Affiliations:** 1 Population Health Sciences Institute, Faculty of Medical Sciences, Newcastle University, Newcastle upon Tyne, UK; 2 National Insitute for Health and Care Research Applied Research Collaboration North East and North Cumbria, Newcastle upon Tyne, UK

**Keywords:** family medicine, health inequities, qualitative research, general practitioners

## Abstract

**Background:**

The Deep End network in the North East and North Cumbria (NENC) was set up to tackle health inequalities in general practice. One aim is to address the inverse care law and improve recruitment of GPs, which is known to be especially challenging in areas of socioeconomic deprivation.

**Aim:**

To explore GP trainees’ experiences and perceptions of working in Deep End or Deprived Area Practices (DE/DAPs) to identify how GP recruitment can be improved.

**Design & setting:**

Qualitative study recruiting 13 doctors training to be GPs from the Northumbria training programme.

**Method:**

Audio-recorded, online, semi-structured interviews and discussion groups were undertaken, transcribed verbatim, and analysed with a grounded theory approach, using a process of thematic analysis.

**Results:**

Overall, seven interviews and two discussion groups (13 participants in total) were conducted. Three themes were identified. The first theme was working in areas of socioeconomic deprivation is challenging but has many advantages. The challenges of working in DE/DAPs were not deterring factors for GP trainees wanting to work in areas of socioeconomic deprivation. The second theme was trainees are willing to work in areas of socioeconomic deprivation but clinical experience is important. Training in DE/DAPs gives trainees the confidence to work in areas of deprivation. Familiarity with a practice also makes them more likely to stay post-training. The third theme was financial incentives are not an important attracting factor but support and development opportunities are. Non-pecuniary measures, such as clinical support and protected time for continuing professional development (CPD), were found to be important.

**Conclusion:**

To improve recruitment to DE/DAPs, investments should be made to increase the opportunities to train in these environments. This can be achieved by supporting more DE/DAPs to become training practices, and providing clinical support and protected time for CPD.

## How this fits in

Previous research has shown working and training as a GP in areas of socioeconomic deprivation is challenging. It has also been shown that practices in such areas are less likely to offer training to prospective GPs. This study adds to this previous research by highlighting the factors GP trainees feel are important when practices in areas of socioeconomic deprivation are recruiting. The study shows the importance of practices offering training to GP trainees, as well as offering clinical support and continuing professional development (CPD) time after training.

## Introduction

General practice recruitment is in crisis in the UK.^1^ Reasons for this are multifactorial but an ever-increasing workload is a major driving factor.^2^ The number of patients per GP has increased by 19% since 2015.^3^ More than 90% of GPs indicate the increased workload has resulted in increased stress,^4^ resulting in one-third considering leaving the profession.^5^ Areas of socioeconomic deprivation are hit hardest. There is an unequal distribution of GPs across the UK, with areas of socioeconomic deprivation having lower numbers of GPs.^6^ A number of potential reasons have previously been proposed for the lack of GPs in areas of socioeconomic deprivation;^7^ for example, working in areas of deprivation is known to be demanding. GPs must manage complex patients with multimorbidities and comorbidities, poor levels of patient engagement, and low health literacy.^8^ Evidence suggests GPs in deprived areas are more stressed, as they attempt to deal with such complex patients in short consultations.^9^ Such challenges contribute to burnout rates being much higher in GPs working in areas of deprivation.^10^

Following in the footsteps of the original Deep End network developed in Scotland,^11^ the Deep End network in the North East and North Cumbria (NENC) was established in 2020 using the same approach to identify general practices serving communities facing extreme and blanket socioeconomic deprivation.^12^ Aims of the network include the bringing together of practices in order to exchange ideas, advocate for change, and develop interventions that improve patient care and the wellbeing of staff.^13,14^ Central to this mission is to increase GP recruitment and retention in areas of socioeconomic deprivation to reverse the inverse care law.^15^

To become a qualified GP in the UK most junior doctors undergo a 3-year training programme. Given most trainees then proceed to working as qualified GPs, they are a key component of the future general practice workforce. There is some evidence to suggest GP trainees are more likely to work in a practice where they have trained.^16,17^ The research has mainly focused on underserved rural GP settings but there is some evidence this is also true in areas of socioeconomic deprivation.^18,19^

There is very limited research looking at improving recruitment of GP trainees in areas of socioeconomic deprivation. A previous study explored the experiences of GP trainees working in areas of socioeconomic deprivation but primarily assessed these experiences from a training perspective.^20^

This study used a qualitative methodology to assess GP trainees’ experiences and perceptions of working in Deep End or Deprived Area Practices (DE/DAPs), with a focus on improving recruitment. It explored the barriers preventing trainees joining such practices and what can be done to overcome such barriers. It also identified GP trainees’ priorities when choosing a practice to work in post-training and what can be done to incentivise recruitment to practices in areas of greater socioeconomic deprivation.

## Method

### Participants

Participants were GP trainees from the Northumbria training programme, covering part of the North East of England. The programme includes >300 trainees at any one time at all stages of training.^21^

### Recruitment

All trainees on the Northumbria training programme were invited to participate via email in April 2022. Consent to email the trainee email distribution list was granted from the Northumbria training programme’s lead training programme director. An invitation email with an attached participant information leaflet was sent to all trainees on the training programme to invite them to participate. Given the training programme has >300 trainees, this ensured recruitment of trainees with a broad range of backgrounds and previous experiences. Convenience sampling of participants known to the author was also used to supplement recruitment.

### Data collection

Participants were asked to respond to the invitation and volunteer to participate. Consent was obtained either electronically or verbally recorded pre-interview. Data were collected through two small group interviews with three participants each, and seven one-to-one interviews depending on participant preference and availability. Each participant only took part in one interview. Interviews followed a semi-structured approach based on a topic guide. MJA, a GP trainee, carried out the interviews between April and June 2022. Interviews were conducted and recorded using Microsoft Teams (version 1.5.00.21463).

### Data analysis

Interviews were auto-transcribed using Microsoft Teams. Transcripts were checked for accuracy and errors by MJA. A grounded theory approach to data analysis was taken using a process of thematic analysis.^22^ All transcripts were read by MJA and JMW to allow for data immersion and familiarity. Data analysis was led by MJA, who conducted close reading and re-reading of the transcripts. A coding framework was developed through a process of constant comparison. As interviews progressed, line-by-line coding was conducted by MJA to compare data and identify new and a-priori codes. NVivo (version 12) software was used to organise and code the data. Connections were then made between these initial codes to form higher-level coding frameworks. These frameworks were discussed and agreed by the whole team before being applied to the transcripts. To ensure rigour, a sample of 15% of the transcripts were independently coded by MJA and JMW. Coding discrepancies were discussed and resolved, including through the refinement of the coding framework. All authors met regularly to discuss theme development and produce the final thematic analysis.

## Results

### Participants

Overall, 13 participants were recruited. A summary of participant characteristics are presented in Table 1. Most participants had experience of working in a deprived area practice and three had worked in a Deep End practice. The Deep End practices in NENC were identified using the same approach as the Scottish Deep End project. This involves using 2019 Index of Multiple Deprivation scores and NHS Digital practice populations by Lower layer Super Output Areas (LSOA). Practices falling into the 10% most deprived in England were identified as Deep End practices.^14^

**Table 1.** Participant characteristics (*N* = 13)

**Characteristic**	***n***
Sex	
Male	2
Female	11
Stage of GP training	
Year 1	2
Year 2	2
Year 3	9
Trained in a Deep End practice	
Yes	4
No	9
Trained in an area of socioeconomic deprivation	
Yes	12
No	1

### Themes

Analysis identified the following three themes: 1) working in areas of socioeconomic deprivation is challenging but has many advantages; 2) trainees are willing to work in areas of socioeconomic deprivation but clinical experience is important; and 3) financial incentives are not an important attracting factor but support and development opportunities are.

#### Working in areas of socioeconomic deprivation is challenging but has many advantages

Participants were asked about their experiences of working in areas of deprivation, including the challenges and positive aspects they encountered.

It was clear trainees experienced challenging clinical scenarios during their time working in areas of deprivation. Examples included complex multimorbidity and drug and alcohol addiction leading to difficult, time-consuming consultations for the GP:

*'People who use drugs regularly don't consult through the normal routes often, they often have a very fixed agenda that I can't meet safely*.*'* (Participant 8, year 3 of training, female, had never worked in a Deep End practice)

Clinical issues combined with complex social issues led to feelings of frustration and powerlessness:

*'I feel that I've been trained to recognise and see how* [social issues] *impacts on health, but I don't feel very equipped to sort that problem out as a root cause and so feel a little bit powerless.'* (Participant 9, year 3 of training, female, had never worked in a Deep End practice)

Frustration was also shared by many of the participants relating to lack of motivation and engagement by patients, which increased the workload and levels of stress. Safeguarding concerns and frequent use of translators were also highlighted as challenges:

*'I found the safeguarding issues are a very, very frequent problem and they are time-consuming. It’s stressful, it feels risky*.*'* (Participant 4, year 3 of training, female, had never worked in a Deep End practice)

*'I have had a lot of consultations via translators and that’s really challenging. That’s a huge thing that I find difficult because you're not getting to the real issue. It feels like at times medicine with a total blindfold on.'* (Participant 10, year 3 of training, female, had never worked in a Deep End practice)

Participants also highlighted the difficulty of managing complex consultations in 10 minutes:

*'I feel as though if you had 15 or 20 minute appointments for these situations you could actually feel as though you could sort of ease into it. Whereas the thought of managing these situations in 10 minutes would fill me with a bit of fear.'* (Participant 12, year 3 of training, female, had worked in a Deep End practice)

However, although challenging, this was not a deterrent for most participants who enjoyed working in a DE/DAP. Many participants highlighted the rewarding nature of the work and the positive relationships with patients who were grateful for their clinical care. This also included acting as a role model for younger patients:

*'Being a role model for young people in deprived areas. So patients can think my GP is actually a real person. I think you could be more instrumental as a practice of improving opportunities for young people.'* (Participant 9, year 3 of training, female, had never worked in a Deep End practice)

Participants also felt they were being allowed to make more pragmatic decisions with less anxiety about making mistakes:

*'There’s a lot of stuff that you kind of have to accept that you can't do anything about or reverse. And for me, I find that easier. It’s clearer to see the things that I can help with.'* (Participant 10, year 3 of training, female, had never worked in a Deep End practice)

Good teamworking was a frequently mentioned experience when participants had worked in DE/DAPs:

*'I think it feels more "teamly" in a way because I think the staff know it’s hard and they understand that the patients are tricky to manage. So you kind of feel like you're in it together and it has a nice feeling.'* (Participant 11, year 2 of training, female, had worked in a Deep End practice)

It was also highlighted that DE/DAPs offered innovative solutions to help their patients. For example, practices were tuned in to the issues facing their patients:

*'We have got quite a lot set up in our practice. It’s not signposting people to apps and websites, it’s directly handing out clothes for the kids and that kind of thing.'* (Participant 10, year 3 of training, female, had never worked in a Deep End practice)

The availability of a wider multidisciplinary team was also widely praised by a number of participants and was often targeted specifically to the needs of the local population:

*'They've got like, mental health link workers and social prescribers and different types of receptionists who they've recruited from the local population. So there’s a couple of people who are* [ethnicity] *and they've got a really unique insight into the population.'* (Participant 13, year 2 of training, male, had never worked in a Deep End practice)

The practices also were good at managing difficult clinical scenarios, such as drug and alcohol addiction, and clinical work was medically interesting:

*'The stuff that I've seen in practice A and B has been medically quite interesting. You see people presenting much later with illness. There’s much more pathology like when I get my list of bloods in the morning.'* (Participant 4, year 3 of training, female, had never worked in a Deep End practice)

#### Trainees are willing to work in areas of socioeconomic deprivation but clinical experience is important

Participants who had no prior experience of working in a DE/DAP were asked about their perceptions of working in such areas and whether they would be willing to work in such practices post-training. Those participants who had worked in deprived areas were asked about their plans post-training and what factors would influence their plans to work in a DE/DAP. All trainees were also asked about their familiarity with the Deep End concept.

Given the challenges of working in a DE/DAP, trainees highlighted the importance of gaining clinical experience of working in such practices. While the majority of the participants had encountered patients experiencing some form of socioeconomic deprivation during their general practice training, one participant had no experience and two participants had limited experience. One of the major concerns was fear of the unknown:

*'My thoughts about going to one of these Deep End practices would be, would I be able to do it? Would I enjoy it? Would I want to commit to a job there having never worked in that type of practice before? So I think I would feel a bit apprehensive about it getting a job there initially.'* (Participant 2, year 3 of training, female, had never worked in a Deep End practice)

This lack of experience was the main driving factor to not wanting to work in a DE/DAP on completion of training:

*'It’s really difficult because I might fit in really well, I've just never done it. So maybe I should just kind of stick with what I know.'* (Participant 2, year 3 of training, female, had never worked in a Deep End practice)

For those trainees who had clinical experience in a DE/DAP, level of deprivation was not an important factor when considering where to work post-training:

*'I don't actually have a preference because I think every population will come with its own challenges and unique things. Ultimately, I see it as that doesn't have a huge bearing on my work. Ultimately the medicine doesn't change dramatically.'* (Participant 13, year 2 of training, male, had never worked in a Deep End practice)

Indeed, the majority of participants stated that they would prefer to work in an area of deprivation:

*'Your aim really should be using your skills in the place where you potentially need it most and that there is a real benefit to doing that and job satisfaction and interest.'* (Participant 12, year 3 of training, female, had worked in a Deep End practice)

The most commonly cited reason for choosing a practice post-training was having already worked in the practice during training. Knowing the team is supportive and being familiar with the practice was important:

*'I've obviously chosen to work where I'm trained and I think a lot of people do the same because they know what they're getting in for and they know the team. That will probably always be the case.'* (Participant 8, year 3 of training, female, had never worked in a Deep End practice)

This was also applicable to working in practices in areas of deprivation:

*'I’ve invested 18 months into getting to grips with all their systems and getting to grips with the complex patients. And I think it would just be a real shame to not carry on building on that really.'* (Participant 12, year 3 of training, female, had worked in a Deep End practice)

Participants highlighted being familiar with the challenges of working in an area of deprivation was important:

*'If you are exposed* [to deprived areas] *it might make you likely to actually apply to those places or not be put off* [by] *it because of the unknown.*' (Participant 4, year 3 of training, female, had never worked in a Deep End practice)

Given the importance of training when choosing a practice to work in, participants were asked about their thoughts on training in a DE/DAP. All participants described the importance of training in both DE/DAPs and affluent practices to gain a wide range of experience:

*'It makes you a more well-rounded doctor. Just because you work in an affluent area doesn't mean you're not going to have patients who have had similar problems to those in a more deprived area that you've worked in before and vice versa. So yeah, I think it should definitely be a compulsory part of training.'* (Participant 5, year 3 of training, female, had never worked in a Deep End practice)

Knowledge of the Deep End concept itself was low among trainees. A number of participants had never encountered the concept of the Deep End, including a participant who was working in a Deep End practice. Other participants understood it related to the level of deprivation and extent of health inequalities but only one participant was aware of its aim to tackle the inverse care law. In light of this, and the need to improve awareness of the concept among trainees, participants were asked about their thoughts on an integrated training programme in the Deep End. This would allow allocated time to work on projects relating to the Deep End or experiencing work in a Deep End practice. Participants thought this would be a good idea but added this would likely appeal to those who already have an interest in socioeconomic deprivation and thus may not improve general recruitment.

#### Financial incentives are not an important attracting factor but support and development opportunities are

Participants were then asked about factors that would encourage them to work in a Deep End practice post-training.

The most important draw to working in a DE/DAP was knowing the practice had adapted to help mitigate the known challenges. Commonly mentioned priorities were 15-minute appointment times, a fixed number of patients per session, and a supportive team. Seven participants stated protected CPD time to develop a special interest would be a big incentive:

*'Opportunities for CPD for development as an individual* [would be a priority]*. That probably comes from having an area of special interest, whether that’s women’s health or education, and that would attract me more to a Deep End practice.'* (Participant 9, year 3 of training, female, had never worked in a Deep End practice)

The majority of participants highlighted the importance of having support or supervision to help the transition from trainee to qualified GP. This supervision did not necessarily need to be formal, rather an informal support network to help alleviate the stress and pressure associated with working in a DE/DAP practice:

*'I guess even more than supervision you need it for the blatant purpose of it just to be like "hey, this made me feel awful" and they'll be like "yeah, don't worry, that’s normal".'* (Participant 11, year 2 of training, female, had worked in a Deep End practice)

Money was not a major driver to attract participants to work in the Deep End. However, given working in Deep End practices is known to be hard, participants would want to feel they are being sufficiently compensated. A number of participants also highlighted that offering high pay or a *‘golden handshake’* may not attract doctors who are likely to stay working in the Deep End:

*'If somebody was only attracted to that job because of the pay then is that the kind of doctor that’s likely to stay long term?'* (Participant 3, year 1 of training, male, had worked in a Deep End practice)

## Discussion

### Summary

Attracting GP trainees to work in areas of socioeconomic deprivation is a key factor in improving the recruitment crisis in DE/DAPs. This study shows that for the majority of trainees the known challenges of working in a DE/DAP are not a deterring factor and, in fact, many trainees are specifically attracted to areas of socioeconomic deprivation. However, the fear of the unknown is a deterring factor for those trainees without prior experience of working in such areas, highlighting the importance of training in DE/DAPs. Training in a DE/DAP not only equips trainees with the skills to work in such practices but also the familiarity with the practice results in a trainee being more likely to stay there on completion of training. This is especially the case if a practice was perceived to be well run, supportive, and with a good teamworking ethos. Incentives, such as protected CPD time, clinical supervision, and at least 15 minutes per appointment, would also encourage trainees to work in a DE/DAP. Such organisational adaptations aim to address heavy workloads and reduce the risk of stress and burnout. In contrast, material gains, such as monetary incentives, were less important. It is also clear from this study that the majority of trainees were not aware of the Deep End concept.

### Strengths and limitations

This qualitative study included trainees with a wide range of experiences. This gave an important insight into the challenges trainees face depending on their experience and what could be done to address such challenges. In addition, the use of GP trainees focused on doctors who are most likely to take up work in DE/DAPs in the foreseeable future. This may help to improve the recruitment crisis in DE/DAPs both in the short and long term. The interviewer’s role as a GP trainee ensured a deep understanding of the concepts raised by the participants allowing for in-depth and detailed discussions.

Difficulties in recruiting medical professionals to research studies is well documented.^23^ This limited the ability to recruit subgroups of participants, such as international medical graduates, which may have enriched the data. It is known that deprived areas are more dependent on GPs who have trained overseas, which has increased in significance post-Brexit.^24^ In addition, as recruitment was in the form of a voluntary response to an email, trainees who already had an interest in health inequalities were more likely to volunteer for the study. This could impact how representative the study population is of all GP trainees.

### Comparison with existing literature

This study confirms that the challenges of working in DE/DAPs previously highlighted^25,26^ are also acknowledged by GP trainees. This complements and expands previous work by Cunningham and Yeoman, which focused on opinions of GP trainees predominantly from a training perspective.^20^ This study briefly highlighted factors that would influence the choice of practice post-training and found heavy workloads leading to burnout and fatigue were concerns when considering working in areas of deprivation. The current study provides more insights into these considerations and also explores potential incentives to working in an area of socioeconomic deprivation. In 2016 a Deep End report was published that assessed GP trainees’ and newly qualified GPs’ experiences of working in the Deep End.^27^ In line with the findings of our study, the report found working in a deprived area was not a deterrent for trainees. It also found having strong nursing support and a good working atmosphere were key factors when choosing a practice to work in. The current study builds on these findings and uses a rigorous qualitative analysis to go into further detail about the experiences and perceptions of GP trainees with respect to working in DE/DAPs in the post-pandemic era. The study focuses on improving recruitment by also exploring trainees’ priorities when considering where to work post-training, an area on which there is limited research.

To our knowledge, this is the first study that considers the experiences of those who have worked in a DE/DAP alongside the perceptions of those trainees who have not. Knowing the perceptions of trainees with no experience is important in being able to understand what might encourage and reassure trainees to consider a DE/DAP role in their career. Previous work has established GPs tend to work where they have trained,^16,17^ including in the context of deprived areas.^18,19^ This study has reaffirmed the importance of this finding from a trainee perspective.

### Implications for research and practice

This study highlights several issues that need to be addressed to tackle the recruitment crisis in DE/DAPs. First, this study provides further evidence that training in DE/DAPs is an important factor for trainees when considering career options on completion of training. It is known that there are fewer training practices in areas of socioeconomic deprivation.^28^ This is owing to a variety of reasons, including high workloads that result in insufficient time to become a training practice.^29^ There needs to be an increase in the number of Deep End practices that are training practices. Not only does the challenging environment improve clinical skills for the doctor but also it ensures they are confident in working in such practices in the future. In addition, training placements in DE/DAPs need to be made a compulsory part of general practice training. This mirrors the opinion of GP trainers in previous work.^30^

In addition, an increased awareness of the Deep End concept during general practice training is needed. We propose this should be taught from medical school through to GP specialty training. It is important trainees are aware of the challenges and benefits of working in areas of deprivation and the opportunities available. Such challenges and benefits need to be acknowledged to improve working conditions for new GPs. Practical measures include allowing at least 15 minutes per appointment, designated CPD time, and clinical supervision and/or mentoring. This is in addition to creating a practice atmosphere that promotes teamwork and a supportive working environment. It is important that these incentives are clearly stated when jobs are advertised. Ideally such jobs should be advertised on a central website that is easy to find, such as the regional Deep End network websites. Importantly, pay does not need to be high but should be in line with other practices. This complements a previous study in underserved rural populations suggesting pecuniary incentives had less impact than non-pecuniary incentives in attracting staff to work in rural areas.^31^

This study’s findings can inform targeted recruitment strategies for practices in areas of socioeconomic deprivation. However, it is clear DE/DAPs would benefit from extra funding to help implement such changes. To accommodate this, there needs to be policy changes on a national level. This could include developing a funding formula that acknowledges level of deprivation.
